# Complexities and actualization: embedding a home-based functional improvement intervention within a Medicaid Waiver

**DOI:** 10.1186/1748-5908-10-S1-A69

**Published:** 2015-08-20

**Authors:** Sarah L Szanton, Sandra Spoelstra, Laura Gitlin

**Affiliations:** 1 Johns Hopkins School of Nursing, Baltimore, MD, 21205, USA; 2 Johns Hopkins School of Public Health, Baltimore, MD, 21205, USA; 3 Michigan State University School of Nursing, East Lansing, MI 48824, USA; 4 Johns Hopkins School of Medicine, Baltimore, MD, 21205, USA

## 

CAPABLE is a multi-component intervention tested through CMMI Innovation funds to improve self-identified functional goals of low-income older adults. CAPABLE components include fixing the home environment and providing up to 10 home visits by occupational therapists and nurses to address functional challenges to self-management. Linking environment and function to health are not traditional home care and Medicaid waiver practices yet potential program outcomes (reduction of functional disability, depression, polypharmacy and nursing home placement) align with Medicaid Waiver goals. Using normalization process theory, we present challenges and opportunities implementing CAPABLE in a new setting: a home health agency in Medicaid Waiver program.

We examine dissemination and implementation dynamics of the intervention, micro-environmental context (staff and agency), macro-environment context (policy, demographic and utilization trends), group conventions, and embedding. Methods include focus groups, semi-structured interviews, joint-home visits, and taped home visits.

Results include the following implementation challenges: health providers misperceive the CAPABLE program as similar to routine care, have scant experience implementing evidence-based care, and require culture shift from clinician-directed to client-directed care. Home repair and modification integrated within the lens of a medically necessary Medicaid requirement is complex, and there is a shortage of occupational therapists.

In contrast, results of the multiple process measures show the following implementation supports: mission-driven staff that became dedicated to the approach once evidence-based strategies helped participants achieve improved function, waiver social workers included in the waiver care model bolster CAPABLE treatment principles, bi-directional communication with State policy makers about scaling CAPABLE to the entire Waiver if health care costs decrease for CAPABLE participants relative to non-CAPABLE Waiver participants.

Findings advance the D&I field by applying rigorous theory and process measures to examine a complex intervention disseminated to a site which includes multiple strengths and challenges to normalization.

Funding for the research was provided by the National Institute on Aging, the Center for Medicare and Medicaid Innovations Center, the John A. Hartford Foundation, the Robert Wood Johnson Foundation, the Blue Cross and Blue Shield of Michigan Foundation, and a Medicaid Match Grant: Translating Evidence into Practice.

